# Colorectal Cancer Screening After Sequential Outreach Components in a Demographically Diverse Cohort

**DOI:** 10.1001/jamanetworkopen.2024.5295

**Published:** 2024-04-16

**Authors:** Clara Podmore, Kevin Selby, Christopher D. Jensen, Wei K. Zhao, Noel S. Weiss, Theodore R. Levin, Joanne Schottinger, Chyke A. Doubeni, Douglas A. Corley

**Affiliations:** 1 Department of Ambulatory Care and Community Medicine, Lausanne University Hospital, Lausanne, Switzerland; 2 Institute of Family Medicine, University of Fribourg, Fribourg, Switzerland; 3 Division of Research, Kaiser Permanente Northern California, Oakland; 4 Department of Epidemiology, University of Washington, Shoreline; 5 Department of Research and Evaluation, Kaiser Permanente Southern California, Pasadena; 6 The Ohio State University Wexner Medical Center, Columbus

## Abstract

**Importance:**

Organized screening outreach can reduce differences in colorectal cancer (CRC) incidence and mortality between demographic subgroups. Outcomes associated with additional outreach, beyond universal outreach, are not well known.

**Objective:**

To compare CRC screening completion by race and ethnicity, age, and sex after universal automated outreach and additional personalized outreach.

**Design, Setting, and Participants:**

This observational cohort study included screening-eligible individuals aged 50 to 75 years assessed during 2019 in a community-based organized CRC screening program within the Kaiser Permanente Northern California (KPNC) integrated health care delivery setting. For KPNC members who are not up to date with screening by colonoscopy, each year the program first uses automated outreach (mailed prescreening notification postcards and fecal immunochemical test [FIT] kits, automated telephone calls, and postcard reminders), followed by personalized components for nonresponders (telephone calls, electronic messaging, and screening offers during office visits). Data analyses were performed between November 2021 and February 2023 and completed on February 5, 2023.

**Exposures:**

Completed CRC screening via colonoscopy, sigmoidoscopy, or FIT.

**Main Outcomes and Measures:**

The primary outcome was the proportion of participants completing an FIT or colonoscopy after each component of the screening process. Differences across subgroups were assessed using the χ^2^ test.

**Results:**

This study included 1 046 745 KPNC members. Their mean (SD) age was 61.1 (6.9) years, and more than half (53.2%) were women. A total of 0.4% of members were American Indian or Alaska Native, 18.5% were Asian, 7.2% were Black, 16.2% were Hispanic, 0.8% were Native Hawaiian or Other Pacific Islander, and 56.5% were White. Automated outreach significantly increased screening participation by 31.1%, 38.1%, 29.5%, 31.9%, 31.8%, and 34.5% among these groups, respectively; follow-up personalized outreach further significantly increased participation by absolute additional increases of 12.5%, 12.4%, 13.3%, 14.4%, 14.7%, and 11.2%, respectively (all differences *P* < .05 compared with White members). Overall screening coverage at the end of the yearly program differed significantly among members who were American Indian or Alaska Native (74.1%), Asian (83.5%), Black (77.7%), Hispanic (76.4%), or Native Hawaiian or Other Pacific Islander (74.4%) compared with White members (82.2%) (all differences *P* < .05 compared with White members). Screening completion was similar by sex; older members were substantially more likely to be up to date with CRC screening both before and at the end of the screening process.

**Conclusions and Relevance:**

In this cohort study of a CRC screening program, sequential automated and personalized strategies each contributed to substantial increases in screening completion in all demographic groups. These findings suggest that such programs may potentially reduce differences in CRC screening completion across demographic groups.

## Key Points

**Question:**

Does colorectal cancer (CRC) screening completion by sequential automated and personalized components differ by demographic characteristics (age, sex, and race and ethnicity)?

**Findings:**

In this cohort study of 1 088 024 screening-eligible individuals, automated and personalized screening program components achieved absolute increases in screening coverage of 29% to 38% and another 11% to 15%, respectively. Personalized components had a higher relative contribution among demographic groups with lower response proportions after automated outreach.

**Meaning:**

These findings suggest that the implementation of automated and personalized components of a CRC screening program was associated with substantial increases in screening completion among all demographic groups assessed in the study, potentially reducing differences in CRC screening completion across demographic groups.

## Teaser

This cohort study examines colorectal cancer (CRC) screening completion by race and ethnicity, age, and sex in an integrated health care delivery setting after sequential universal automated outreach and personalized outreach.

## Introduction

Comparisons of colorectal cancer (CRC) incidence and mortality by race and ethnicity in the US have shown consistent disparities, with mortality being highest among non-Hispanic Black (hereinafter Black) individuals.^1^ Factors leading to lower uptake of CRC screening among some populations are an important contributor to these disparities. In 2006 to 2008, Kaiser Permanente Northern California (KPNC) initiated an organized population-based multistep screening program based primarily on mailed fecal immunochemical test (FIT) kits for KPNC members not up to date with screening. By 2018, implementation of this program was associated with the virtual elimination of differences in CRC incidence and death between Black members and non-Hispanic White (hereinafter, White) members, concordant with relatively high screening levels in both groups.^2^

Evidence from randomized clinical trials shows that FIT outreach by mail or in person, patient navigation, patient education, and patient reminders can all increase uptake of CRC screening.^3^ However, these interventions require very different levels of resources, from inexpensive mailed reminders to personalized high-touch navigators.^4,5^ With heterogeneity in the distribution of social and structural barriers in the US, strategies to deliver CRC screening in the community-based setting may differ in effectiveness across racial and ethnic groups and other demographic groups.^6^ In addition, how individuals respond to different portions of complex, multicomponent interventions remains poorly defined.^6^ There are minimal data for some racial and ethnic groups, such as American Indian or Alaska Native individuals and Native Hawaiian or Other Pacific Islander individuals, in the extant literature. Such knowledge can be highly valuable for those seeking to establish outreach programs, by estimating the relative effectiveness of different interventions. To inform these questions, this study evaluated the response to individual screening program strategies in a large, organized CRC screening program according to race and ethnicity and other demographic groups.

## Methods

### Study Design and Participants

This cohort study included KPNC members who were eligible for CRC screening at the beginning of 2019. Kaiser Permanente Northern California is a large, integrated health care delivery organization whose membership reflects the region’s US Census demographics, including racial and ethnic groups.^7^ Its active membership includes approximately 4.5 million people, or more than 1% of the US population. The KPNC Institutional Review Board approved this study and waived the requirement for individual informed consent because the protocol posed no more than minimal risk to participants. The study followed the Strengthening the Reporting of Observational Studies in Epidemiology (STROBE) reporting guideline. Information about KPNC members was obtained from the KPNC electronic health record and administrative databases. Race and ethnicity was captured by self-report at health plan enrollment, patient visit registration, and, for some patients, by employers during employer-based enrollment. Available categories included American Indian or Alaska Native, Asian, Black, Hispanic, Native Hawaiian or Other Pacific Islander, White, and other race or ethnicity (self-identified as a race or ethnicity other than the options provided). These categories were mutually exclusive, implying that the Hispanic category could include members who identified as being of any race (ie, Asian, Black, or White). Approximately 4% of members in the cohort were excluded from analyses owing to missing race and ethnicity data. We obtained data on sex (self-reported as man or woman), age at the date of cohort entry, and Charlson Comorbidity Index score^8^ for each member. We used the percentage of persons in the US Census tract aged 25 years or older who had graduated from high school using 2006 to 2010 as an estimate of socioeconomic status (SES) based on the 5-year estimates from the American Community Survey. This measure is correlated with the overall socioeconomic conditions of neighborhoods.^9,10^ We categorized the SES measure into quartiles such that US Census tracts with the lowest percentage of high school graduates were classified in the fourth quartile.

### Screening Program

**Figure 1.**
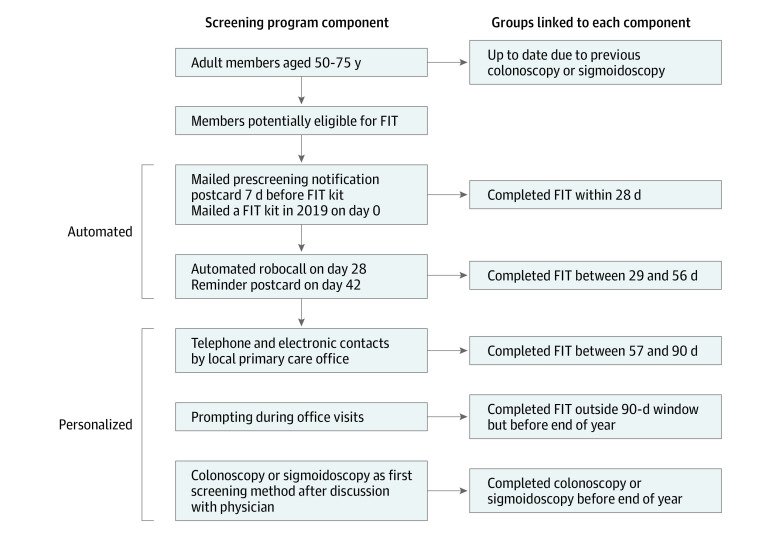
Kaiser Permanente Northern California Colorectal Cancer Screening Program Process via Automated and Personalized Components and Corresponding Measures of Screening Completion FIT indicates fecal immunochemical test.

Detailed descriptions of the KPNC CRC screening program have been published previously^11,12^ and are summarized here and in Figure 1. The program’s goal is to screen all screening-eligible members by the end of each calendar year via FIT or colonoscopy. Colonoscopy is provided based on patient or physician request and, for persons not up to date by other methods, a FIT kit is mailed annually. Screening outreach through 2019 started the year a given member turned 51 through age 75 years in accordance with the Healthcare Effectiveness Data and Information Set measurement approach.^13^ The screening program uses a series of automated and personalized approaches that are delivered sequentially based on the screening status at any point in time.

Individuals who have undergone colonoscopy within the last 10 years or sigmoidoscopy within the last 5 years or who have completed an FIT in the current calendar year are considered up to date. Members are eligible for screening outreach if they are not up to date with screening by colonoscopy or sigmoidoscopy on December 31 of the prior year or if their next FIT is due in the current calendar year.

#### Automated Components

Eligible members are mailed a prescreening notification postcard, followed 7 days later by a FIT kit that includes a letter with the photograph and signature of the member’s primary care physician (PCP), pictorial FIT instructions, and a prepaid return envelope. Members who do not return the test receive an automated telephone call 28 days after FIT kit mailing and a postcard reminder 42 days after mailing. These automated components include culturally tailored messages, created with stakeholder input, for Black and Hispanic individuals.

#### Personalized Components

Eight weeks after FIT kit mailing, lists of members who have not completed screening are made available to their PCP’s office for further local follow-up. Medical assistants then make telephone calls or send personalized electronic messages or mailings (as possible) approximately 8 to 13 weeks after FIT kit mailing. In addition, local quality program staff make telephone calls or send electronic messages to members not yet up to date with screening. Throughout the year, eligible members who attend office visits and are identified as not up to date with screening are offered the FIT. Colonoscopy is also available upon patient or physician request.

#### Laboratory Processing of FITs

Whether received by mail or in person, all FITs completed at home are returned to a designated central laboratory for initial quality control reviews. Tests with no date or illegible information are evaluated by the laboratory’s client services department, after which (when appropriate) the required test information is manually entered into the laboratory database, an order is placed, and a label is generated for subsequent automated processing. If the test is not suitable for processing, a new FIT kit is mailed to the member with an explanation of the error. Tests are analyzed with an automated OC-Sensor Diana analyzer (Polymedco Inc), with a cutoff of greater than 20 mg hemoglobin/g stool for a positive result. Patients with a positive test result are contacted for follow-up colonoscopy to complete the screening process.

### Statistical Analysis

For this study, we computed the proportion of members who were up to date with screening in each racial and ethnic subgroup at different stages of the screening process. We compared absolute differences in the proportions of members completing screening using logistic regressions with racial and ethnic groups as independent variables, with White members as the reference group, as this was the largest subgroup. Differences across these subgroups were assessed using the χ^2^ test; *P* < .05 (2-tailed) was considered statistically significant. Screening completion at different stages of the process was also stratified by age groups and sex to assess differences in screening participation by these 2 factors. Members who self-reported as other gender or had missing data for sex were excluded from sex-stratified analyses. Sensitivity analyses assessed differences across racial and ethnic groups restricted to individuals aged 50 to 54 years with 1 to 5 years of KPNC membership and also in the fourth quartile of SES. This was done because a larger proportion of Hispanic individuals and Native Hawaiian or Other Pacific Islander individuals were aged 50 to 64 years compared with individuals in other racial and ethnic groups. We performed additional sensitivity analyses for each calendar year between 2014 and 2018 to determine whether the results from 2019 were stable over time. We also computed the proportion of members with a positive FIT result who had follow-up colonoscopy at 180 days.^14^

All analyses were conducted using SAS, version 9.4 (SAS Institute, Inc). Data analyses were performed between November 2021 and February 2023 and completed on February 5, 2023.

## Results

Among 1 088 024 eligible KPNC members, we identified 1 046 745 for whom race and ethnicity data were available. The cohort had a mean (SD) age of 61.1 (6.9) years as of January 1, 2019, and comprised 557 390 women (53.2%) and 489 321 men (46.7%); 2 members self-reported as other gender, and 32 members were missing data for sex. Baseline characteristics of the cohort are presented in Table 1. Participants identified as American Indian or Alaska Native (0.4%), Asian (18.5%), Black (7.2%), Hispanic (16.2%), Native Hawaiian or Other Pacific Islander (0.8%), White (56.5%), or other race or ethnicity (0.5%). White members were older and less likely to be female than Asian or Black members. Black, Hispanic, and Native Hawaiian or Other Pacific Islander members had lower levels of high school educational attainment on the SES measure than the other racial and ethnic groups. Comorbidity scores were higher among Black and Native Hawaiian or Other Pacific Islander members.

**Table 1.** Baseline Characteristics of the 2019 KPNC Cohort by Race and Ethnicity^a^

Characteristic	Race and ethnicity						
	American Indian or Alaska Native (n = 3670 [0.4])	Asian (n = 194 032 [18.5])	Black (n = 75 116 [7.2])	Hispanic (n = 169 146 [16.2])	Native Hawaiian or Other Pacific Islander (n = 7871 [0.8])	White (n = 591 755 [56.5])	Other^b^ (n = 5155 [0.5])
Age, y							
50-64	2597 (70.8)	134 796 (69.5)	51 544 (68.6)	127 047 (75.1)	5845 (74.3)	366 805 (62.0)	3665 (71.1)
65-75	1073 (29.2)	59 236 (30.5)	23 572 (31.4)	42 099 (24.9)	2026 (25.7)	224 950 (38.0)	1490 (28.9)
Sex							
Women	1910 (52.0)	105046 (54.1)	42400 (56.5)	88885 (52.6)	3935 (50.0)	312273 (52.8)	2941 (57.1)
Men	1760 (48.0)	88985 (45.9)	32711 (43.6)	80253 (47.5)	3936 (50.0)	279462 (47.2)	2214 (43.0)
High school educational attainment, SES quartile^c^							
1	659 (18.0)	51 828 (26.7)	9615 (12.8)	18 592 (11.0)	1038 (13.2)	177 179 (29.9)	1226 (23.8)
2	834 (22.7)	48 573 (25.0)	15 244 (20.3)	29 763 (17.6)	1679 (21.3)	164 496 (27.8)	1305 (25.3)
3	986 (26.9)	48 565 (25.0)	20 502 (27.3)	41 993 (24.8)	2276 (28.9)	145 324 (24.6)	1393 (27.0)
4	1187 (32.3)	44 867 (23.1)	29 509 (39.3)	78 532 (46.4)	2864 (36.4)	103 712 (17.5)	1218 (23.6)
Missing	4 (0.1)	199 (0.1)	246 (0.3)	266 (0.2)	14 (0.2)	1044 (0.2)	13 (0.3)
Duration of KPNC membership, y							
1-5	959 (26.1)	45 419 (23.4)	13 669 (18.2)	40 271 (23.8)	1924 (24.4)	111 084 (18.8)	1000 (19.4)
6-10	818 (22.3)	40 138 (20.7)	13 486 (18.0)	37 033 (21.9)	1661 (21.1)	115 953 (19.6)	939 (18.2)
>10	1893 (51.6)	108 475 (55.9)	47 961 (63.8)	91 842 (54.3)	4286 (54.5)	364 718 (61.6)	3216 (62.4)
CCI score							
0	2141 (58.3)	120 666 (62.2)	39 994 (53.2)	101 068 (59.8)	4109 (52.2)	371 094 (62.7)	2996 (58.1)
1	729 (19.9)	36 414 (18.8)	14 336 (19.1)	33 108 (19.6)	1611 (20.5)	105 821 (17.9)	1078 (20.9)
2	390 (10.6)	17 920 (9.2)	8748 (11.6)	17 225 (10.2)	933 (11.9)	54 614 (9.2)	512 (9.9)
3	410 (11.2)	19 032 (9.8)	12 038 (16.0)	17 745 (10.5)	1218 (15.5)	60 226 (10.2)	569 (11.0)
FIT completion the year prior^d^	1500 (58.7)	90 523 (69.7)	30 265 (61.8)	72 275 (61.1)	3349 (59.0)	251 736 (66.9)	2161 (64.7)

Abbreviations: CCI, Charlson Comorbidity Index; FIT, fecal immunochemical test; KPNC, Kaiser Permanente Northern California; SES, socioeconomic status.

^a^ Values are presented as No. (%) of participants.

^b^ Includes members who self-identified as a race or ethnicity other than the options provided.

^c^ Categorized into quartiles such that US Census tracts with the lowest percentage of high school graduates were classified in the fourth quartile.

^d^ Among members eligible for screening (%).

**Figure 2.**
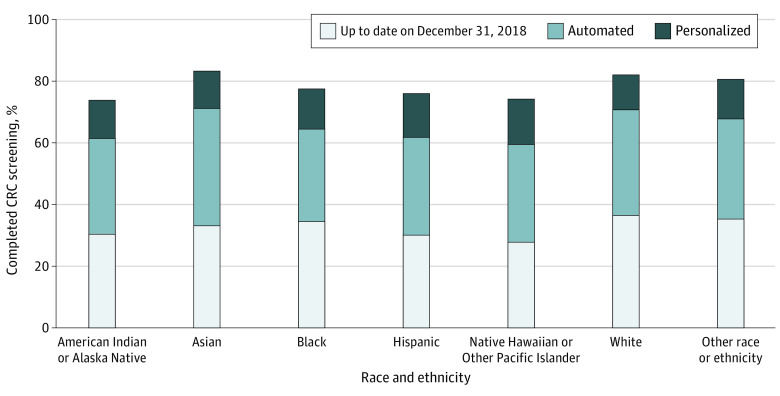
Colorectal Cancer (CRC) Screening Completion via Automated and Personalized Components by Race and Ethnicity Among Kaiser Permanente Northern California Members in 2019 Up to date indicates the proportion of members not eligible for a fecal immunochemical test (FIT) that year because, as of January 1, they had completed a colonoscopy within the last 9 years or sigmoidoscopy within the last 5 years. Automated indicates the proportion of members who completed a FIT within 56 days of FIT kit mailing; they received a prescreening notification postcard by mail (7 days prior), FIT kit by mail, robocall (28 days after kit mailing), and postcard reminder (42 days after kit mailing). Personalized indicates the proportion of members who completed a FIT, colonoscopy, or sigmoidoscopy outside of the 56-day window after FIT kit mailing, presumably linked to a telephone call, electronic message, in-clinic reminder, or contact with a health care professional.

Figure 2 presents the proportion of members up to date with CRC screening by December 31, 2018, and following the automated and personalized components of the screening process during the 2019 calendar year by race and ethnicity. Absolute differences in screening completion across racial and ethnic groups compared with White members (reference group) are detailed in Table 2. White members (36.4%) were most likely to be up to date with screening before the beginning of the year compared with Black (34.8%), Hispanic (30.1%), and Asian (33.1%) members; Native Hawaiian or Other Pacific Islander members (27.9%) were the least likely to be up to date. These differences were statistically significant for all subgroups compared with White members, except for individuals of other race or ethnicity (35.2%). After the screening process, the proportion of members up to date with screening by the end of 2019 ranged from the lowest value of 74.1% among American Indian or Alaska Native members to the highest of 83.5% among Asian members (with 77.7%, 76.4%, 74.4%, and 82.2% for Black, Hispanic, Native Hawaiian or Other Pacific Islander, and White members, respectively; Figure 2).

**Table 2.** Absolute Differences in Colorectal Cancer Screening Completion by Race and Ethnicity According to Sequential Components of the Screening Process

Screening completion	Race and ethnicity												
	American Indian or Alaska Native		Asian		Black		Hispanic		Native Hawaiian or Other Pacific Islander		White	Other^a^	
	Absolute difference^b^	*P* value	Absolute difference	*P* value	Absolute difference	*P* value	Absolute difference	*P* value	Absolute difference	*P* value		Absolute difference	*P* value
Already up to date by December 31, 2018	−6.0 (−7.6 to −4.5)	<.001	−3.4 (−3.6 to −3.1)	<.001	−1.6 (−2.0 to −1.3)	<.001	−6.3 (−6.6 to −6.1)	<.001	−8.6 (−9.7 to −7.5)	<.001	Reference	−1.2 (−2.5 to 0.1)	.07
Automated	−3.5 (−5.0 to 1.9)	<.001	3.6 (3.3 to 3.8)	<.001	−5.0 (−5.4 to −4.7)	<.001	−2.7 (−2.9 to −2.4)	<.001	−2.7 (−3.8 to −1.7)	<.001	Reference	−1.9 (−3.2 to −0.6)	<.001
Personalized (including colonoscopy)	1.4 (0.4 to 2.4)	<.001	1.2 (1.0 to 1.3)	<.001	2.1 (1.9 to 2.4)	<.001	3.2 (3.0 to 3.4)	<.001	3.5 (2.8 to 4.3)	<.001	Reference	1.5 (0.7 to 2.4)	<.001
Total screened by December 31, 2019	−8.1 (−9.3 to −6.8)	<.001	1.4 (1.2 to 1.6)	<.001	−4.5 (−4.8 to 4.2)	<.001	−5.8 (−6.0 to −5.8)	<.001	−7.8 (−8.6 to −6.9)	<.001	Reference	−1.6 (−2.6 to −0.5)	<.001

^a^ Black and White refer to non-Hispanic Black and non-Hispanic White, respectively. Other race or ethnicity includes members who self-identified as a race or ethnicity other than the options provided.

^b^ Absolute differences are presented as % (95% CI).

The proportion of individuals who completed CRC screening increased substantially across all racial and ethnic subgroups after delivery of both the automated and personalized components. Asian members and White members (38.1% and 34.5% absolute increases) had the highest completion proportions and Black members (29.5% absolute increase) had the lowest completion proportions to the initial automated components (with absolute increases of 31.1%, 31.9%, and 31.8% for American Indian or Alaska Native, Hispanic, and Native Hawaiian or Other Pacific Islander members).

The additional completion proportions following subsequent personalized approaches were highest among Hispanic members (14.4%) and Native Hawaiian or Other Pacific Islander members (14.7%) (with 12.5%, 12.4%, 13.3%, and 11.2% for American Indian or Alaska Native, Asian, Black, and White members, respectively). The differences in CRC screening after delivery of both the automated and personalized components were statistically significant for all subgroups compared with White members. The use of colonoscopy as a first method of screening was similar across all racial and ethnic groups (<2.0%). Members who identified as being of other race or ethnicity had similar screening proportions at each step as White members.

The proportions of members up to date with CRC screening after automated and personalized components were similar between men and women (Table 3). Across different age groups, older members were substantially more likely to be up to date with CRC screening at both the beginning (>40.0% for those aged 66-75 years) and end (>80.0% for those aged 66-75 years) of 2019 relative to younger members (20.8% vs 72.1%, respectively, for those aged 50-55 years). Personalized components had a higher relative contribution to the completion proportions in younger members than in older age groups (ranging from 17.9% among members aged 50-55 years from personalized components to 10.6% of those aged 61-65 years and 7.2% of those aged 71-75 years).

**Table 3.** Colorectal Cancer Screening Completion by Automated and Personalized Approaches by Age and Sex Among KPNC Members in 2019

Screening characteristic	Age, y										Sex^a^			
	50-55		56-60		61-65		66-70		71-75		Men		Women	
	No. (%)	Cumulative %	No. (%)	Cumulative %	No. (%)	Cumulative %	No. (%)	Cumulative %	No. (%)	Cumulative %	No. (%)	Cumulative %	No. (%)	Cumulative %
Already up to date on January 1, 2019 (prior colonoscopy or sigmoidoscopy)	56 697 (20.8)	20.8	79 705 (33.1)	33.0	82013 (37.6)	37.6	83 962 (43.9)	43.9	59 561 (48.1)	48.1	170 702 (34.9)	34.9	191 222 (34.3)	34.3
Eligible	215 584 (79.2)	NA	161 488 (67.0)	NA	136 059 (62.4)	NA	107 463 (56.1)	NA	64 213 (51.9)	NA	318 619 (65.1)	NA	366 168 (65.7)	NA
Automated														
Completed within 28 d	60 579 (22.3)	43.1	58 199 (24.1)	57.2	57 839 (26.5)	64.1	53 047 (27.7)	71.6	33 699 (27.2)	75.4	122 038 (24.9)	59.8	141 320 (25.4)	59.7
Completed in 28-56 d	30 490 (11.2)	54.3	23 902 (9.9)	67.1	19 699 (9.0)	73.2	14 273 (7.5)	79.0	7701 (6.2)	81.6	44 236 (9.0)	68.9	51 825 (9.3)	69.0
Total	91 069 (33.5)	NA	82 101 (34.0)	NA	77 538 (35.6)	NA	67 320 (35.2)	NA	41 400 (33.5)	NA	166 274 (34.0)	NA	193 145 (34.7)	NA
Personalized														
Completed in 56-90 d	10 296 (3.8)	58.1	7167 (3.0)	70.1	5140 (2.4)	75.5	3410 (1.8)	80.8	1664 (1.3)	82.9	12 635 (2.6)	71.4	15 039 (2.7)	71.7
FIT outside 90-d window	33 213 (12.2)	70.3	18 754 (7.8)	77.8	14 073 (6.5)	82.0	9880 (5.2)	86.0	5334 (4.3)	87.2	36 563 (7.5)	78.9	44 690 (8.0)	79.7
Colonoscopy or sigmoidoscopy	4989 (1.8)	72.1	3995 (1.7)	79.5	3856 (1.8)	83.7	3162 (1.7)	87.6	1823 (1.5)	88.7	8081 (1.7)	80.6	9744 (1.8)	81.4
FOBT	156 (0.1)	72.1	139 (0.1)	79.6	132 (0.1)	83.8	119 (0.1)	87.7	103 (0.1)	88.8	302 (0.1)	80.6	347 (0.1)	81.5
Total	48 654 (17.9)	NA	30 055 (12.5)	NA	23 201 (10.6)	NA	16 571 (8.7)	NA	8924 (7.2)	NA	57 581 (11.8)	NA	69 820 (12.5)	NA
Not screened by December 31, 2019	75 861 (27.9)	NA	49 332 (20.5)	NA	35 320 (16.2)	NA	23 572 (12.3)	NA	13 889 (11.2)	NA	94 764 (19.4)	NA	103 203 (18.5)	NA
Screened by December 31, 2019	196 420 (72.1)	NA	191 861 (79.6)	NA	182 752 (83.8)	NA	167 853 (87.7)	NA	109 885 (88.8)	NA	394 557 (80.6)	NA	454 187 (81.5)	NA
Total	272 281 (100)	NA	241 193 (100)	NA	218 072 (100)	NA	191 425 (100)	NA	123 774 (100)	NA	489 321 (100.0)	NA	557 390 (100)	NA

Abbreviations: FIT, fecal immunochemical test; FOBT, fecal occult blood test; KPNC, Kaiser Permanente Northern California; NA, not applicable.

^a^ Subset numbers may not sum to the total number of members because 2 members self-reported as other gender and 32 members had missing data for sex.

In sensitivity analyses restricted to members aged 50 to 54 years, those with a membership duration of 1 to 5 years and those in the fourth quartile of SES showed similar trends across racial and ethnic groups in the response proportions to each component of the screening process (eTable in Supplement 1). When we repeated the main analysis by calendar year between 2014 and 2018, the differences between racial and ethnic groups were similar over time.

Between 3.3% (Asian members) and 4.7% (Native Hawaiian or Other Pacific Islander members) of FIT results were positive. Across all racial and ethnic groups, approximately 80.4% to 82.9% of members completed follow-up colonoscopy within 180 days of a positive test result except for Native Hawaiian or Other Pacific Islander members, among whom 72.9% completed follow-up within 180 days.

## Discussion

This cohort study of more than 1 million individuals in an organized CRC screening program addressed the relative importance of established evidence-based delivery approaches by race and ethnicity, age, and sex. The findings suggest that automated and personalized screening program components each contributed substantially to high overall proportions of up-to-date status for CRC screening across all racial and ethnic groups in the analysis. Personalized outreach had slightly higher relative contributions among Black, Hispanic, and Native Hawaiian or Other Pacific Islander individuals and among younger patients (aged 50-54 years).

Meta-analyses of randomized clinical trials demonstrate the effectiveness of active outreach as well as patient navigation for maximizing up-to-date status for CRC screening.^3,15^ The current study provides reassurance that similar approaches, as used in the current study, can improve FIT uptake across racial and ethnic subgroups, while finding that the magnitude differs somewhat across selected key demographic groups. Disparities in uptake of CRC screening have been consistently reported in the literature, with higher screening levels among Asian and White populations.^16^ The current study also enabled analyses of groups that are typically understudied (eg, American Indian or Alaska Native individuals and Native Hawaiian or Other Pacific Islander individuals).

Our findings suggest that personalized screening approaches further boosted completion of screening in all members, beyond universal automated approaches, with a larger contribution among Black, Hispanic, and Native Hawaiian or Other Pacific Islander members and among younger members. One may expect that differences in racial and ethnic groups might be partially explained by differences in age distribution, SES, or membership duration, but we observed similar trends in restricted analyses by these subgroups, suggesting this is not the case. Outcomes associated with personalized approaches delivered to younger members are particularly relevant because since 2021, the US Preventive Services Task Force has also recommended CRC screening among individuals aged 45 to 49 years (grade B recommendation).^17^

A logical next question for health systems considering implementing the KPNC screening approach is the associated cost with organized outreach. The collection of detailed cost information was beyond the scope of this study. Estimates from similar interventions vary widely. For example, a series of intensive navigation interventions implemented by the Centers for Disease Control and Prevention had estimated costs between $1000 and $3500 USD per person screened, including colonoscopy costs.^18^ A larger intervention in Washington State using FIT mailings and reminder telephone calls had a total cost just under $40 per FIT kit returned.^19^

Our findings suggest that being approached by a known and trusted individual from a PCP’s office may enhance completion of screening, particularly among individuals with lower initial responses to automated outreach. However, the exact component of supplemental outreach that influenced these results cannot be directly assessed.^20^ Direct contact from practice staff or a PCP could address concerns and barriers to screening. In randomized clinical trials, FIT outreach seems equally effective across all populations, whereas patient navigation appears to be relatively more effective in settings with a larger proportion of individuals from historically disadvantaged populations.^3^

We also observed that the integrated health care system studied resulted in comparable proportions of follow-up for positive FIT results at 180 days (80.4% to 82.9%) in all racial and ethnic groups except for Native Hawaiian or Other Pacific Islander individuals (72.9%), based on 166 positive FIT results in 2019, and with similar overall proportions of follow-up observed in previous years.^21^ Further research should aim to obtain a better understanding of possible specific barriers faced by Native Hawaiian or Other Pacific Islander individuals to completing the screening process and of measures to address these barriers.

### Limitations

Our primary limitation is that this study was observational, such that causality cannot be inferred in the link between differential responses to outreach components and demographic categories. Because of the successive nature of the screening program components, response to screening invitation was time based and cannot be attributed clearly to an individual component. Similarly, we were unable to determine whether personalized outreach was more effective than simply repeating automated outreach because we did not have a comparison group. However, anecdotal experience suggests that repeated automated reminders, such as robocalls, may be negatively perceived by members. The observed differences between racial and ethnic groups may be due to unmeasured confounders, such as political orientation or geographic differences. More in-depth data may be needed. Finally, Kaiser Permanente uses a specific informatics infrastructure in electronic health records to support CRC screening. With increasing adoption of digital health technology, similar systems of reminders and tracking can be replicated in other settings but the capacity to do so may be limited in resource-constrained settings.

## Conclusions

In this cohort study of a multicomponent CRC screening program, automated and personalized components were associated with increases in screening in all racial and ethnic groups, within a setting that has demonstrated marked decreases in CRC incidence and mortality with increased screening. Despite the limitations described, these results can inform clinicians and other settings seeking to increase CRC screening and to decrease disparities in screening uptake, cancer incidence, and cancer deaths.^2^ Future research should include the collection of cost information in large-scale programs and qualitative studies to better understand how participant opinions and preferences regarding outreach differ between subgroups.

## Notes

Supplement 1. **eTable.** Colorectal Cancer Screening Completion by Automated and Personalized Approaches by Race and Ethnicity Among Kaiser Permanente Northern California Members in 2019

Supplement 2.
Data Sharing Statement
