# Supplementary material for: Colorectal Cancer Screening After Sequential Outreach Components in a Demographically Diverse Cohort
Source: JAMA Netw Open. 2024 Apr 16;7(4):e245295. doi: 10.1001/jamanetworkopen.2024.5295 (PMC11022110; doi:10.1001/jamanetworkopen.2024.5295)
Supplement: Supplement 2. — Data Sharing Statement [file jamanetwopen-e245295-s002.pdf]

Data Sharing Statement

Podmore. Colorectal Cancer Screening After Sequential Outreach Components in a Demographically Diverse Cohort. *JAMA Netw Open*. Published April 16, 2024.
doi:10.1001/jamanetworkopen.2024.5295

Data
Data available: No

Additional Information
Explanation for why data not available: Dataset can be available for collaborative efforts by contacting Dr Douglas Corley at douglas.corley@kp.org.
